# Bioelectronic modulation of carotid sinus nerve to treat type 2 diabetes: current knowledge and future perspectives

**DOI:** 10.3389/fnins.2024.1378473

**Published:** 2024-04-05

**Authors:** Silvia V. Conde, Joana F. Sacramento, Ciro Zinno, Alberto Mazzoni, Silvestro Micera, Maria P. Guarino

**Affiliations:** ^1^iNOVA4Health, NOVA Medical School, Faculdade de Ciências Médicas, Universidade NOVA de Lisboa, Lisbon, Portugal; ^2^The BioRobotics Institute Scuola Superiore Sant’Anna, Pontedera, Italy; ^3^ciTechCare, School of Health Sciences Polytechnic of Leiria, Leiria, Portugal

**Keywords:** bioelectronic medicine, carotid body, carotid sinus nerve, neuromodulation, type 2 diabetes

## Abstract

Bioelectronic medicine are an emerging class of treatments aiming to modulate body nervous activity to correct pathological conditions and restore health. Recently, it was shown that the high frequency electrical neuromodulation of the carotid sinus nerve (CSN), a small branch of the glossopharyngeal nerve that connects the carotid body (CB) to the brain, restores metabolic function in type 2 diabetes (T2D) animal models highlighting its potential as a new therapeutic modality to treat metabolic diseases in humans. In this manuscript, we review the current knowledge supporting the use of neuromodulation of the CSN to treat T2D and discuss the future perspectives for its clinical application. Firstly, we review in a concise manner the role of CB chemoreceptors and of CSN in the pathogenesis of metabolic diseases. Secondly, we describe the findings supporting the potential therapeutic use of the neuromodulation of CSN to treat T2D, as well as the feasibility and reversibility of this approach. A third section is devoted to point up the advances in the neural decoding of CSN activity, in particular in metabolic disease states, that will allow the development of closed-loop approaches to deliver personalized and adjustable treatments with minimal side effects. And finally, we discuss the findings supporting the assessment of CB activity in metabolic disease patients to screen the individuals that will benefit therapeutically from this bioelectronic approach in the future.

## 1 Introduction

Metabolic diseases, like metabolic syndrome and type 2 diabetes (T2D), affect millions of people worldwide, being considered epidemics by the World Health Organization (WHO) (World Health Organization, 2023). The number of people affected by T2D will exceed half a billion by 2040 (IDF, 2021). T2D is characterized by peripheral insulin resistance, abnormal hepatic glucose metabolism and progressive pancreatic beta cell failure. Glucose control in T2D deteriorates progressively over time and, after failure of lifestyle modifications such as diet and exercise, there is a need of a new intervention with glucose-lowering agents every 3–4 years, on average, in order to obtain/retain good glycemic control (Davies et al., 2022). Despite combination therapy and/or insulin treatment, a sizable proportion of individuals remain poorly controlled (World Health Organization, 2023), highlighting the need for novel and personalized therapeutic approaches. Apart from diabetes itself, comorbidities like cardiovascular disease, kidney disease, neuropathy, blindness, and lower-extremity amputation are significant causes of morbidity and mortality among people with diabetes resulting in a huge burden in healthcare systems and on society (IDF, 2021; World Health Organization, 2023). The financial impact of diabetes is significant, with health expenditures dedicated to its treatment and complication prevention estimated at approximately €150 billion in the European Union in 2021 (IDF, 2021) and $412.9 billion in the United States in 2022 (Parker et al., 2024). Therefore, innovative therapeutic approaches are necessary. One potential focus is on modulating the carotid body (CB) and its sensitive nerve, the carotid sinus nerve (CSN), which are emerging as promising targets for treatment.

## 2 Role of carotid body chemoreceptors and of the carotid sinus nerve in the pathogenesis of metabolic diseases

**Figure 1:**
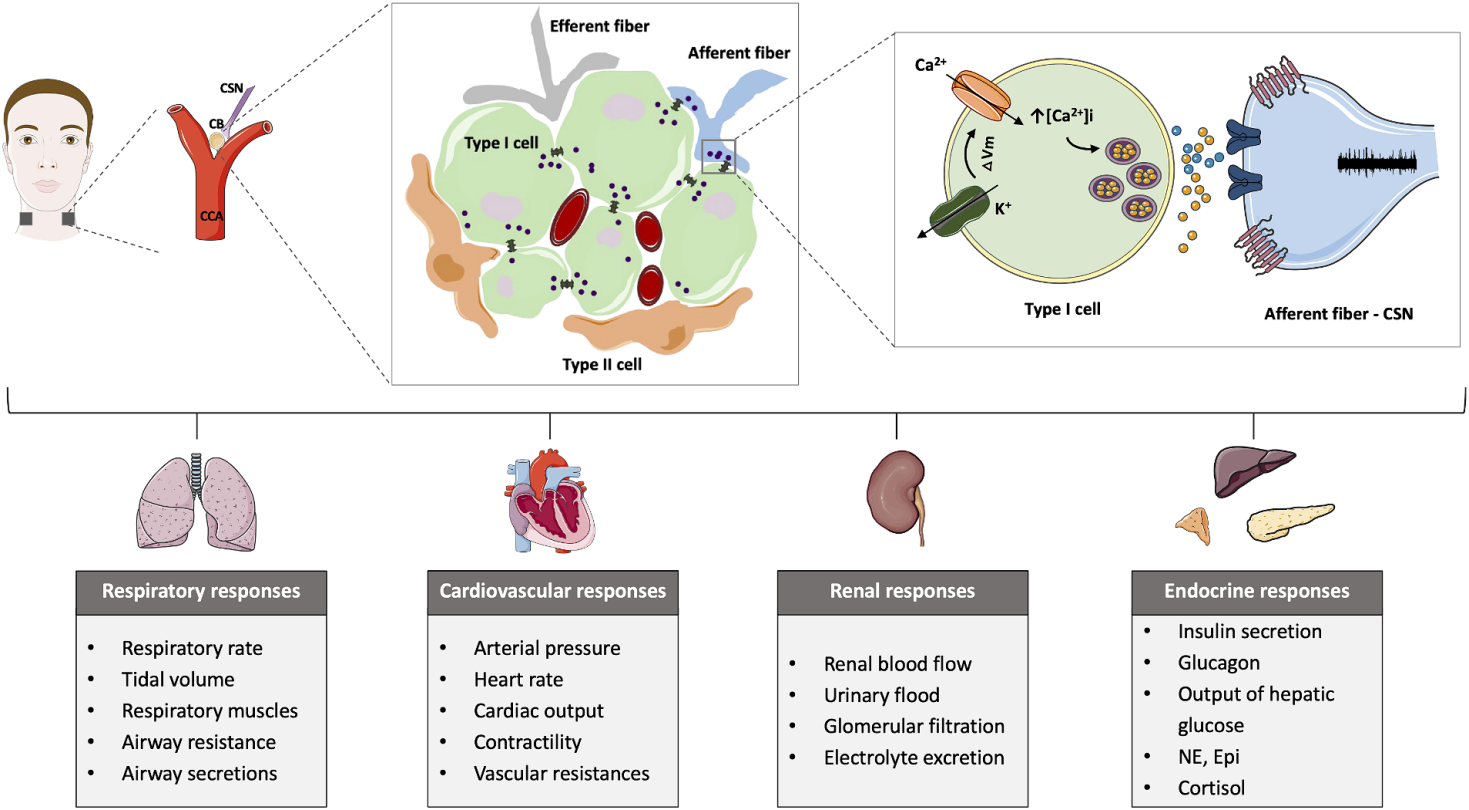
Schematic representation of the carotid body (CB) location, morphology, mechanisms of chemotransduction and the different chemoreflexes elicited by the CB activation. The CB is located bilaterally in bifurcation of the common carotid artery (CCA) and is formed by type I cells, the chemosensory unit of the CB, that are organized around capillaries and are surrounded by type II cells. The stimulation of the CB, for example, in response to low oxygen levels, induces the closure of K^+^ channels that promotes membrane depolarization. The alteration in the membrane potential opens Ca^2+^ channels and increases the intracellular calcium, triggering the release of neurotransmitters that act postsynaptically at the carotid sinus nerve (CSN) to increase its activity. The CSN activity is integrated in the brain stem and produce respiratory, cardiovascular, renal and endocrine responses.

The CB is a sensor of arterial gases, such as oxygen and carbon dioxide, also detecting pH and temperature (Gonzalez et al., 1994; Kumar and Prabhakar, 2012). The main cellular component of the CB is the type I cells or chemoreceptor cells that are derived from the neural crest having electrical excitable properties (for a revision see Iturriaga et al., 2021). These cells contain several neurotransmitters, such as catecholamines (dopamine (DA) and norepinephrine), acetylcholine, serotonin, ATP, as well as other mediators such as neuropeptides – substance P and enkephalins- and adenosine, among others (Conde et al., 2014; Iturriaga et al., 2021; Figure 1). Type I cells are surrounded by type II (sustentacular) cells of glial origin (Pardal et al., 2007) and are enervated by the CSN which activity is integrated in the nucleus tractus solitarii (Iturriaga et al., 2021). There is a general consensus that upon stimulation, in type I cells, there is the closure of K^+^ channels eliciting cell depolarization, with the consequent opening of Ca^2+^ voltage operated channels, Ca^2+^ entry and the increase in intracellular free Ca^2+^ that leads to exocytosis and neurotransmitter release (Gonzalez et al., 1994; Iturriaga et al., 2021; Figure 1). These neurotransmitters act on the CSN to inhibit or to stimulate CSN electrical activity. The CSN is composed of A-fibers (myelinated) and C-fibers (unmyelinated), being the conduction velocity higher in A-fibers (De Castro, 1951; Gonzalez et al., 2014; Porzionato et al., 2019). The A-fiber population comprised afferents of approximately 2/3 of CB chemoreceptors and 1/3 of carotid sinus baroreceptors origin (Fidone and Sato, 1969). In contrast, 54% of the C-fiber population observed in the cat CSN are from autonomic origin, 29% are baroreceptors and 17% are chemoreceptors (Fidone and Sato, 1969). Regarding type II cells, they have been previously considered to have merely a supportive role, however, nowadays it is known that the CB possess adult neural stem cells (or a subpopulation of them). It has been shown that these type II cells contribute to neurogenesis *in vivo* in response to prolonged sustained and intermittent hypoxia by acting in paracrine signaling and contribute to the overactivation of these organs in several pathological conditions (Pardal et al., 2007; Platero-Luengo et al., 2014; Caballero-Eraso et al., 2023). In addition to the function of arterial gases sensor, the CB has a polymodal nature capable of responding to numerous stimuli, such as leptin or pro-inflammatory cytokines, like IL-1β and TNF-α (Kumar and Prabhakar, 2012; Sacramento et al., 2020). More recently, a function as a metabolic sensor has been postulated (Conde et al., 2018; Sacramento et al., 2020), based on its ability to sense alterations in arterial blood glucose, insulin (Ribeiro et al., 2013; Baby et al., 2023), leptin (Ribeiro et al., 2018; Caballero-Eraso et al., 2023) and GLP-1 levels (Pauza et al., 2022). In line with this assumption Conde and co-workers have proposed that early CB dysfunction is involved in the pathogenesis of metabolic diseases (Conde et al., 2014, 2017, 2018). In 2013, Ribeiro and collaborators proposed that the CB regulates peripheral insulin sensitivity through sympathetic nervous system (SNS) modulation, based on the observations that bilateral resection of the CSN, the CB-sensitive nerve, prevented the development of metabolic dysfunction induced by hypercaloric diets in rats (Ribeiro et al., 2013). Apart from preventing the dysmetabolic states induced by hypercaloric diets intake, CSN resection reversed insulin resistance and glucose intolerance and attenuated the weight gain in animal models of dysmetabolism (Ribeiro et al., 2013; Sacramento et al., 2017, 2018; Melo et al., 2022). Trying to go deep into the mechanisms behind the beneficial effects of CSN denervation on metabolism we found that CSN resection restored glucose uptake in the liver and adipose tissue and insulin signaling in skeletal muscle and adipose tissue (Sacramento et al., 2017). Furthermore, CSN resection normalized several features of sympathetic overactivation: increased plasma and adrenal medulla catecholamine levels, increased heart rate variability in the low-frequency zone (LF) as well as the ratio between low and high frequencies of heart rate (LF/HF) (Ribeiro et al., 2013; Sacramento et al., 2017). This CB-driven sympathetic overactivation and its disappearance upon CSN resection was also confirmed in dysmetabolic animal models through electrophysiological recordings made in the upper cervical chain (Cracchiolo et al., 2019a,b). Altogether the data clearly suggest that the CB controls metabolic efferent-end-organs through the sympathetic nervous system. In agreement, CB stimulation induces insulin secretion (Petropavlovskaya, 1953) and arterial and supra-hepatic vein glucose concentration (Alvarez-Buylla and de Alvarez-Buylla, 1988) effects that are probably mediated by the sympathetic nervous system as suggested by Kim and Polotsky (2020).

Endorsing the hypothesis that CB dysfunction contributes to metabolic diseases, are the preclinical and clinical observations that animals submitted to hypercaloric diets (Ribeiro et al., 2013) and individuals with prediabetes (Cunha-Guimaraes et al., 2020) exhibit increased basal ventilation. In humans, an increased CB chemosensitivity was also assessed by the Dejours test, which measures the decrease in basal ventilation produced by 100%O_2_, which was significantly higher in the prediabetes population and correlated with insulin resistance and fasting insulin levels (Cunha-Guimaraes et al., 2020). More recently, Lis and collaborators (Lis et al., 2022) showed that individuals with prediabetes and T2D exhibited an exaggerated peripheral chemoreflex sensitivity, assessed by the hypoxic ventilatory response (HVR) that was associated with the severity of the disease. In animal models with metabolic pathology, increased peripheral chemosensitivity was associated with: (1) an increase in CB weight; (2) an increase in the percentage of type I cells; (3) raised activity of the enzyme tyrosine hydroxylase, as well as increased release of catecholamines by the CBs (Ribeiro et al., 2013; dos Santos et al., 2018) and (4) increased CSN activity (Ribeiro et al., 2018; Cracchiolo et al., 2019a,b). In agreement with these results, other authors had previously observed that also in T2D patients, the size of the CBs increased by approximately 25% compared to non-diabetic volunteers (Cramer et al., 2014). In another experimental perspective, but still in line with these findings, Paleczny et al. described that overweight/obese men exhibit an increased blood pressure response to hypoxia, mediated by peripheral chemoreceptors, although respiratory and heart rate responses to hypoxia remain unaltered. This effect was associated with hyperinsulinemia and insulin resistance but not with hyperleptinemia (Paleczny et al., 2016).

Consolidating the set of preclinical and clinical data supporting that CB dysfunction is involved in the genesis of metabolic diseases, it was shown that the functional suppression of CB activity using hyperbaric oxygen therapy, that delivers 100%O_2_ at a pressure higher than the atmospheric pressure, improves fasting glucose levels and post-prandial glucose tolerance in T2D patients (Vera-Cruz et al., 2015). Altogether the data lead to the conclusion that CB dysfunction is involved in the etiology of metabolic diseases due to overactivation of the SNS and that the CB or its sensitive nerve, the CSN, are therapeutic targets for treating prediabetes and T2D. The current state of the art considers that CB and CSN dysfunction occur in very early stages of dysmetabolism and that its timely screening and diagnosis may represent a powerful tool for disease prevention. Metabolic diseases are a significant public health problem. More resources are needed for the detection of risk factors that are not only associated with lifestyles but may have genetic and hereditary characteristics that, if confirmed, require earlier and more careful monitoring.

On the other hand and given that there is great difficulty in reducing the prevalence of risk factors for metabolic disease, secondary prevention may be justified, i.e., to carry out procedures for a diagnosis of CB overactivation as early as possible for identification of putative candidates for therapeutic CB-specific modulation.

## 3 Bioelectronic neuromodulation of carotid sinus nerve to treat type 2 diabetes

Conventional treatment for metabolic diseases is based on behavioral and pharmacological interventions aiming to maintain glycemic control, to mitigate complications and to assure quality of life. However, while anti-diabetic monotherapy fails to meet the needs of all patients, anti-diabetic drug combination therapy comes with multiple challenges including the complexity of dosing regimens, side effects, and socioeconomic barriers to access, which can lead to medication non-adherence and may not be suitable for all patients (Sugandh et al., 2023). Moreover, conventional treatment for T2D is grounded on the premise of one-size-fits-all, which has shown limitations in addressing the diverse nature of the disease. In recent years, personalized medicine has emerged as a transformative solution, tailoring treatment plans based on individual patients’ unique physiological and pathological characteristics.

Bioelectronic medicine represents a pioneering approach that intertwines cutting-edge technology with personalized medicine (Famm et al., 2013; Birmingham et al., 2014; Adameyko, 2016; Peeples, 2019; Gonzalez-Gonzalez et al., 2024). This concept emerged in the last decade as a new class of treatment consisting of modulation of pathological electrical signaling patterns in the nervous system using implantable miniaturized devices (Famm et al., 2013; Birmingham et al., 2014; Adameyko, 2016; Peeples, 2019; Gonzalez-Gonzalez et al., 2024). It is an emerging and exciting area of research as it aspires to a selective, personalized and patient-adaptive therapy with minimal adverse effects (Famm et al., 2013; Birmingham et al., 2014; Pavlov and Tracey, 2019; Gonzalez-Gonzalez et al., 2024). This therapeutic approach may also have high acceptance among patients, as it may, in the future, require minimally invasive procedures while offering less interference in daily activities compared to current therapeutic options (Famm et al., 2013; Birmingham et al., 2014; Pavlov and Tracey, 2019; Gonzalez-Gonzalez et al., 2024). Bioelectronic medicine has been tested in animal models for several diseases: bladder underactivity (Chen et al., 2021), bladder overactivity (Fletcher, 2020) resistant and chronic hypertension (Annoni et al., 2019), asthma (Mehmed, 2015), and in clinical studies for chronic auto-immune disorders, such as rheumatoid arthritis (Levine et al., 2014; Koopman et al., 2016; Zachs et al., 2019; Genovese et al., 2020), inflammatory bowel disease (Bonaz et al., 2016; Yasmin et al., 2022), and systemic lupus erythematosus, among others. Stimulation of several autonomic nerves has been applied for treating these and other diseases in clinical studies as well, e.g., vagus nerve stimulation was used for obesity (Morton et al., 2016); stimulation of the carotid sinus for hypertension (Heusser et al., 2010) and heart failure (Zile et al., 2020; splenic nerve stimulation is currently under clinical studies for the treatment of rheumatoid arthritis)^1^ and sacral nerve stimulation for inflammatory bowel disease (Pikov, 2023) among others. Moreover, the concept of electrical modulation of the CB chemoreceptors have also been tested for several conditions, including sepsis (Santos-Almeida et al., 2017; Falvey et al., 2020), colitis (Soares et al., 2023), sudden death syndrome (Biggs et al., 2022) and T2D (Sacramento et al., 2018; Table 1). This holds particular significance given that the unilateral and bilateral surgical ablation of the CBs, already explored as a therapeutic approach in certain pathologies characterized by the overactivation of the CB and SNS, such as hypertension (Narkiewicz et al., 2016) and heart failure (Niewiński et al., 2013), may present several drawbacks due to adverse effects associated with the loss of physiological functions mediated by the CB (Conde, 2018). These adverse effects can include a diminished response to hypoxia (Timmers et al., 2003), reduced sensitivity to CO2 (Timmers et al., 2003; Dahan et al., 2007), alterations in physiological responses to physical exercise (Forster et al., 1983; Forster and Pan, 1994; Paton et al., 2013), and fluctuations in blood pressure (Paton et al., 2013; Fudim et al., 2015). CSN resection may also have significant limitations in a setting of long-term therapy, as there is neuronal regeneration of CSN fibers over time (Zapata et al., 1976).

**Table 1:** Carotid sinus nerve (CSN) acute and chronic neuromodulation in different pathologies and animal models.

Authors	Effect on CSN activity	Electrical parameters	Animal model	Parameters evaluated	Outcomes
Santos-Almeida et al. (2017)	Acute stimulation	1 mA, 30 Hz, 1 ms for 1 h	Rat	Inflammatory cytokines	Prevented lipopolysaccharide-induced systemic inflammation in conscious rats, which involved both sympathetic and parasympathetic activation.
Sacramento et al. (2018)	Chronic blocking – 1 week	Rectangular pulses, 2 mA, 50 kHz	Rat	Ventilation	Decreased basal ventilation and inhibited the ventilatory responses to hypoxia in control animals.
	Chronic blocking – 9 weeks			Insulin sensitivity, glucose tolerance	Restored insulin sensitivity and glucose tolerance in conscious type 2 diabetic rats, an effect that was reversible.
Fjordbakk et al. (2019)	Acute stimulation	Symmetric biphasic pulses (width 1,000 μs), 0.2–4 mA, 20 Hz for 20 s	Pig	Tidal volume, respiratory rate, mean arterial blood pressure, heart rate	Increased tidal volume and decreased respiratory rate, mean arterial pressure and heart rate, in anesthetized pigs.
	Acute blocking	6 mA, 20 kHz for 30 s			Block evoked chemo-afferent responses to chemical hypoxia, namely tidal volume, in anesthetized pigs.
Falvey et al. (2020)	Acute stimulation	Rectangular biphasic pulses 100 μs: 200 μA, 5 Hz or 400 μA, 10 Hz, 2 min	Mouse	Inflammatory cytokines, corticosterone, breath rate and blood pressure	Increased breath rate and decreases arterial blood pressure in anesthetized mice. Attenuated inflammation induced by lipopolysaccharide through the increase in corticosterone production that act on the glucocorticoid receptor of myeloid immune cells.
	Chronic stimulation	200 μA, 5 Hz, 0.1 ms for 5 min, twice per day for 3 days		Inflammatory cytokines	Protected against lipopolysaccharide-induced endotoxemia shock in conscious mice.
Ribeiro et al. (2020)	Chronic stimulation	5–3 V; 1 ms; 30 Hz; 10 min per day during 10 days	Rat	Inflammatory response, gene expression and alveolar bone loss associated with periodontitis	Attenuates alveolar bone loss and inflammation in experimental periodontitis
Conde et al. (2022b)	Acute stimulation	Rectangular pulses, 2 mA, 20 kHz for 10 s	Rat	Respiratory frequency, heart rate, mean blood pressure	Increased respiratory frequency and decreased blood pressure in anesthetized rats.
	Acute blocking	Rectangular pulses, 2 mA, 50 kHz for 1 min			Abolished the ventilatory hypoxic response in anesthetized rats but did not alter mean blood pressure and heart rate.
Biggs et al. (2022)	Acute stimulation	Biphasic square waves with a pulse width and inter-pulse delay of 1 ms, 7.5 Hz for 10s. Amplitudes varying from 500 μA intervals in the 1–5 mA interval	Rat	Blood pressure, heart rate, respiration, seizures, ictal mammalian diving reflex	Stimulation of the CB and CSN significantly reduce the likelihood of death from ictal mammalian diving reflex and increases the number of ictal mammalian diving reflex challenges that an animal can survive
Soares et al. (2023)	Acute stimulation	1.5–3 V, 1 ms duration, frequency of 30 Hz for 10 min	Rat	Blood pressure, colitis scores and histological parameters, pro and anti-inflammatory cytokines	Reduction in colonic tissue lesions, colitis scores, and histopathologic parameters associated with colitis. Reduction in colonic mucosa pro-inflammatory cytokine and increased anti-inflammatory interleukin-10 in a colitis model

**Figure 2:**
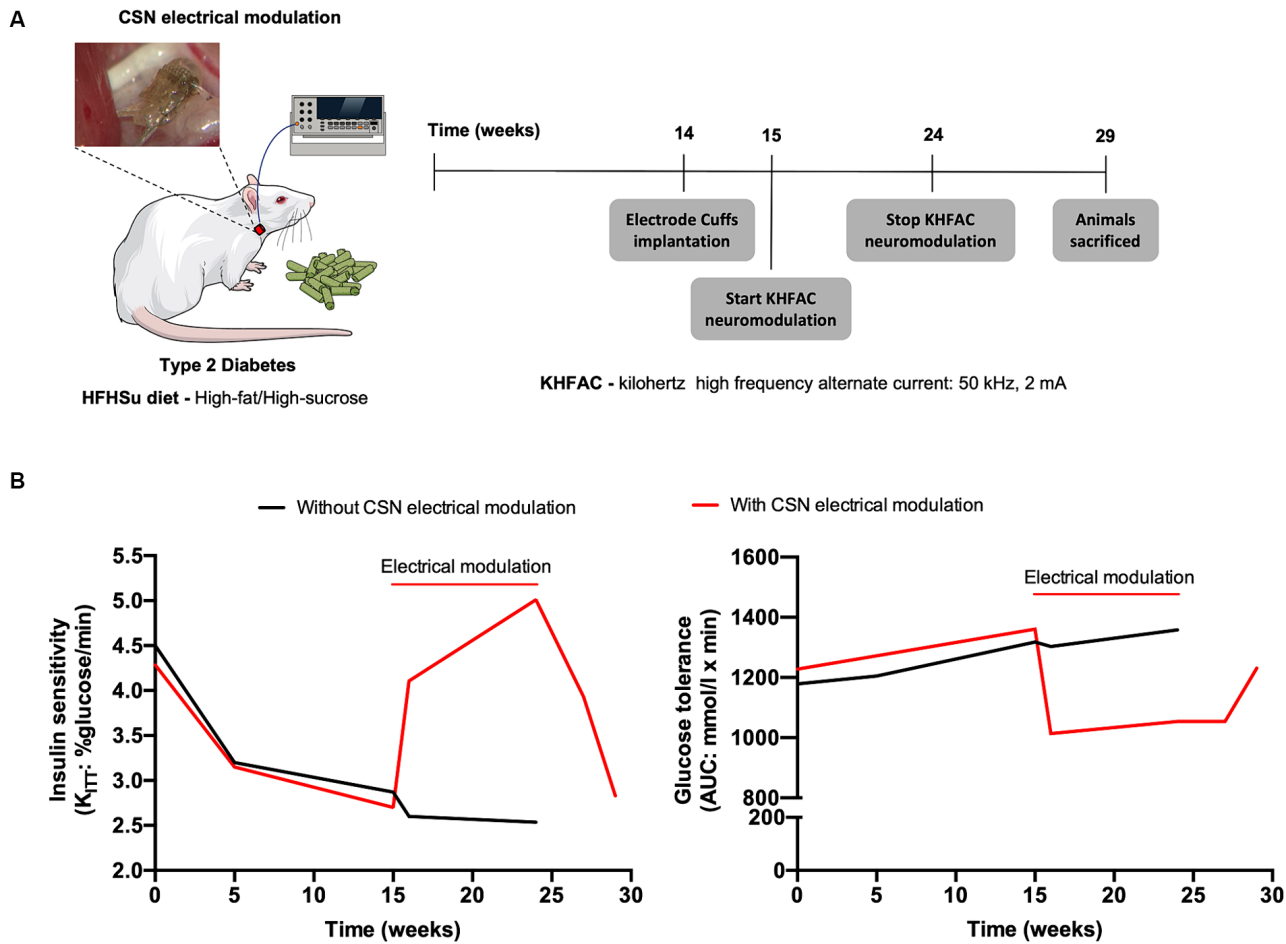
Carotid sinus nerve (CSN) electrical modulation reverses insulin resistance and glucose intolerance in type 2 diabetes (T2D) rats. **(A)** Schematic illustration of the protocol of the study in which rats fed during 14 weeks with high fat-high sucrose (HFHSu) were implanted with electrode cuffs and submitted to 9 weeks of continuous kilohertz high frequency alternate current stimulation (KHFAC, 50 kHz, 2 mA). Animals were followed for 5 weeks after KHFAC cessation. **(B)** Impact of 9 weeks chronic electrical modulation on insulin sensitivity (left panel) and on glucose tolerance (right panel) in HFHsu animals. Note that after KHFAC cessation the animals start to recover the dysmetabolic phenotype.

In the context of T2D, the modulation of the CSN with kilohertz high frequency alternating current (KHFAC, 50KHz, 2 mA), i.e., continuous electrical blockade of the CSN, was shown to have the ability to restore insulin sensitivity and glucose tolerance in T2D rats after 1 week, effects that were maintained during the 9 weeks of blockade (Figure 2 and Table 1; Sacramento et al., 2018). These beneficial effects of electrical modulation of the CSN were also shown to be reversible, as 5 weeks after cessation of KHFAC, insulin resistance and glucose intolerance returned to pathological values (Figure 2 and Table 1; Sacramento et al., 2018). These results indicate that electrical modulation of the CSN is a viable therapeutic strategy for modulating CSN activity in metabolic diseases.

KHFAC is usually referred to charge-balanced alternating current with frequency ranging from 1 to 100 kHz (Kilgore and Bhadra, 2014). It produces an immediate blockade of the nerve as the action potentials are arrested when they reach the depolarizing charge field of the electrode (Kilgore and Bhadra, 2014; Patel et al., 2017). Several molecular mechanisms have been postulated to be responsible for this arrest, with the sodium channel inactivation hypothesis being the most plausible one, supported by considerable experimental evidence (for a review see Kilgore and Bhadra, 2014). For example using the frog sciatic nerve/gastrocnemius muscle preparation, the mammalian axon model and computer simulation of KHFAC it has been shown that KHFAC resulted in an increased inward sodium current compared to the outward potassium current, leading to a dynamic membrane depolarization of a number of nodes under the electrode (Kilgore and Bhadra, 2004; Bhadra et al., 2007), that leads to the inactivation of about 90% of the sodium channels in the node directly under the electrode (Ackermann et al., 2011). Moreover, KHFAC is rapidly reversible upon cessation of block (see, e.g., Kilgore and Bhadra, 2014; Patel et al., 2017; Sacramento et al., 2018). One characteristic that might compromise the use of KHFAC in clinical setting is an initial nerve activation, before complete block occurs, called the “onset response.” Several methods are being developed to avoid/diminish the onset response, as for example the use of ramp-amplitudes (Vrabec et al., 2019); the combination of direct current and KHFAC (Eggers et al., 2021); optimization of KHFAC waveforms to produce closed-state Na^+^ channel inactivation (Yi and Grill, 2020); as well as the use of higher KHFAC amplitudes and frequencies (Gerges et al., 2010). In agreement, we have been able to block CSN activity specifically at 40–50 kHz, 1–2 mA without causing an abnormal physiological onset response (Table 1; Sacramento et al., 2018).

Apart from being tested in rodents the high potential of KHFAC as a strategy for modulating CSN activity was also confirmed in the pig (Fjordbakk et al., 2019; Table 1), thus boosting the translation of the electrical modulation of the CSN for its clinical use. The authors showed that KHFAC modulation of the CSN in this animal model was able to block the NaCN-evoked chemo-afferent responses. However, the CSN not only transmits information that comes from the CB, but also information from the baroreceptors located in the carotid sinus, which are involved in the rapid adjustment of blood pressure (Marshall, 1994; Marcus et al., 2014). The blockade of CSN activity may hinder the baroreceptor adjustments in blood pressure but we have recently observed that acute KHFAC neuromodulation of the CSN (50Khz, 2 mA) does not modify either basal blood pressure or blood pressure responses evoked by hypoxic hypoxia or even heart rate (Conde et al., 2022a,b). These findings indicate that the chosen high frequency stimulation conditions are without significant adverse effects on the cardiovascular system. Nevertheless, the effects of KHFAC on cardiovascular parameters were only tested acutely and information on the impact of chronic KHFAC on these parameters is still lacking. Furthermore, apart from the baroreceptor information conveyed by the CSN, the CB responds to other important physiological stimuli like hypoxia and hypercapnia (Gonzalez et al., 1994). Circumventing the interference that blockade of the CSN may have with the abovementioned homeostatic responses may be possible through the characterization of the types of fibers/electrical patterns involved in dysmetabolism signaling and by the use of closed-loop feed-back control systems to correct the altered pathological neural patterns on demand.

## 4 Advances in CSN neural decoding to achieve personalized and real-time closed-loop neuromodulation

The CSN has become an interesting neuromodulation target for the purpose of treating metabolic disorders since it was first identified as a metabolic sensor (Conde et al., 2018). Hence, the decoding of the CSN electrical activity could provide access to the perception of whole-body glucose uptake by the nervous system rather than to the actual value of glucose blood glucose. Knowledge of glucose-related electrical activity in the CSN might be particularly important for detecting a dysfunctional metabolic state. In fact, it has been shown in rats that the electroneurogram of the CSN contains information related with the metabolic status of the animals. In particular, in animals submitted to hypercaloric diets, the electroneurogram of the CSN shows a shift in its frequency spectrum towards the high frequency (Cracchiolo et al., 2019a,b), that is associated with an increased activity of the SNS found in metabolic dysfunction states (Canale et al., 2013; Lambert et al., 2015). A solution to inhibit the activity of the CSN is the application of the KHFAC, a technique that exploits the properties of high-frequency simulation to perform reversible nerve blockade. To achieve this purpose, Sacramento et al. (2018) employed cuff electrodes with KHFAC (50 kHz, 2 mA) in T2D rats to restore insulin sensitivity and glucose tolerance (Figure 2 and Table 1). This effect was observed in both acute and chronic settings with physiological parameters returning to baseline condition when KHFAC was removed, corroborating the reversibility of this approach. All these neuromodulation studies followed an “open loop” approach and generated important data sets to advance knowledge in the field. However, the key point was that the high-frequency shift observed in the electroneurogram of the CSN in high fat-high sucrose (HFHSu) animals (Cracchiolo et al., 2019a,b) was not constant over time, being particularly strong during alterations in metabolic status. These high-frequency shifts observed during alterations in the metabolic status could be ideally exploited to design a closed loop neural stimulation occurring only during abnormal frequency shifts. This kind of approach would not only increase effectiveness, but also minimize off-target effects due to stimulation of fibers that involve more than one physiological stimulus.

A critical aspect is the hardware supporting the brain-machine interface for reading and stimulating the nerves (Shokur et al., 2021). Until now, most of the studies on the impact of CSN high-frequency neuromodulation employed two-contact cuff electrodes to deliver the electrical current (Sacramento et al., 2018; Fjordbakk et al., 2019). One main limitation of the two-contact cuff electrodes is their lack of selectivity in the stimulation as they activate all nerve fibers at the same time. To obtain unidirectional activation of the fibers, often four-contacts cuff electrodes are used: on the central contact high frequency stimulation is applied (25–40 kHz) to obtain inhibition, while on the distal contact low frequency is applied (15–20 Hz), to obtain directionality (Payne et al., 2022). This approach might be of interest to modulate CSN function, as it will have the ability to block the afferent fibers, that transmit the information to the central nervous system, and to increase the activity of the efferent fibers, that come from the central nervous system to inhibit CB activity (Gonzalez et al., 1994). Another approach to obtain directional and selective stimulation is the use of intraneural electrodes. These devices are designed to be inserted inside the nerve, with their active elements (where there is the exchange of charge between the electrode and the tissue) in close contact with the nerve fibers. Pioneering studies in the vagus nerve have shown that with these devices it is possible to selectively decode neuronal activity from autonomic nerves within the different active sites of the electrode with temporal precision (Micera et al., 2010; Ghazavi et al., 2020; Vallone et al., 2021). This could inspire the use of these devices also to modulate CSN activity aiming the control of peripheral organs both in open and/or closed-loop fashion.

Data on vagal nerve stimulation (VNS) may also pave the way for an effective stimulation of CSN in terms of selectivity. Several studies (Meyers et al., 2016; Stauss et al., 2018; Payne et al., 2020, 2022) have highlighted the effect of VNS of afferent and efferent fibers when stimulating the cervical and abdominal region of the vagus nerve. Stimulation of the afferent branches increases blood glucose, proposed to be due to inhibition of insulin secretion (Stauss et al., 2018), while stimulation of the efferent ones results in a decrease of glycemia, together with a recovery of glucose tolerance and reduction of insulin levels (Meyers et al., 2016; Payne et al., 2022). Therefore, we anticipate the need of similar studies, as those performed at the vagus nerve, with tests of different degrees and paradigms of high-frequency stimulation on the CSN and also with the support of modeling approaches (Romeni et al., 2020), that have already been proved effective to design optimal stimulations for peripheral nerves, aiming to design a closed loop algorithm for selective neuromodulation of the CSN.

## 5 Predicting the efficacy of CSN electrical modulation in type 2 diabetes through screening of CB activity

Among metabolic diseases, diabetes is high prevalent one worldwide, however, about 44% of patients are asymptomatic, which leads to a later diagnosis of the disease and, consequently, increases the risk of developing complications (World Health Organization, 2023). Thus, it is essential to search for new forms of early diagnosis for the adoption of preventive measures and to reverse early stages of the disease, particularly in this pathology that remains asymptomatic until advanced stages. The assessment of CB chemosensitivity has recently emerged as a biomarker in prediabetes and T2D. Assessment of CB chemosensitivity through the classical Dejour’s test clearly shown that individuals with prediabetes exhibit increased CB chemosensitivity that correlates with insulin levels and insulin resistance (Cunha-Guimaraes et al., 2020). A close relationship between CB chemosensitivity and metabolic control in humans, was supported by the study of Vera-Cruz et al. (2015) in where hyperoxia - that is known to block CB function - in hyperbaric conditions, improved glucose homeostasis in T2D patients. The diagnosis of CB function may have predictive value for the risk of developing metabolic diseases in humans (Dejours, 1962; Cunha-Guimaraes et al., 2020), however there are no established devices that accurately and non-invasively assess CB activity allowing early diagnosis of CB overactivation and follow up monitoring. Preliminary studies are being conducted with a prototype of the CBmeter, a device that evaluates, in a minimally invasive way, the activity of CBs, to demonstrate the proof of concept, aiming at future clinical validation (Lages et al., 2021; Conde et al., 2022a). This device was designed to assess the usefulness of monitoring physiological variables closely associated with CB activity as early predictors of insulin resistance. The first prototype consists of a combination of several sensors, already marketed, that were connected to determine heart rate (HR), respiratory rate (RR), peripheral oxygen saturation and interstitial glucose in a synchronous manner (Guarino et al., 2022). The protocol of the prototype testing included assessment of the abovementioned variables in response to challenge tests, namely, acute hyperoxia, as described by Dejours, and an innovative mixed meal challenge test (Lages et al., 2022), grounded on the findings that the CBs respond to insulin variation in the bloodstream. Thus, HR, RR, SpO2 and interstitial glucose are the biosignals monitored by the CBmeter and the prototype was tested in two groups of participants: prediabetes patients and a non-prediabetic control group. In the later, the CBmeter readings were validated against gold standard methodologies for accuracy evaluation. The major finding of the pilot feasibility study was that the decrease in RR upon an hyperoxic challenge was noticeably less pronounced in the metabolically healthy control group than in the prediabetes group, corroborating the data from Cunha-Guimaraes et al. (2020) that showed that prediabetes patients exhibit an increased chemosensitivity. After the administration of the mixed standard meal, the response was not so pronounced since this provocation test does not represent an acute stimulus for CB as the administration of oxygen.

**Figure 3:**
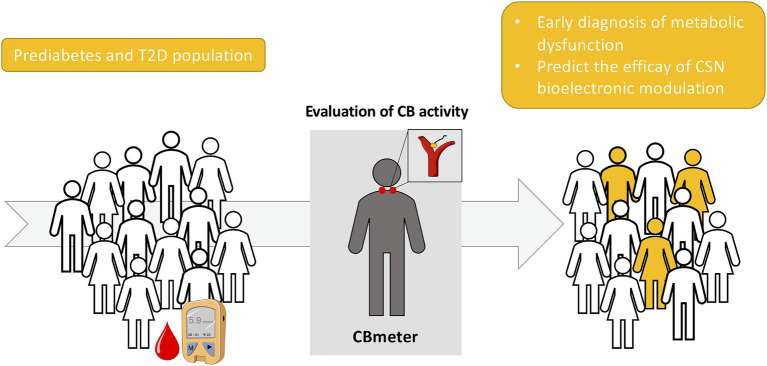
Schematic representation of the screening of carotid body (CB) activity as a biomarker for an early detection of metabolic dysfunction as well as a predictor of the efficacy of carotid sinus nerve (CSN) bioelectronic modulation for the treatment of type 2 diabetes (T2D).

These findings support that assessment of CB activity may be a useful routine screening in a context of preventive medicine, allowing early detection of dysmetabolism long before clinical features are observed like obesity, T2D and hypertension. Moreover, the CBmeter may also represent a useful tool to select the prediabetic or T2D individuals that will benefit the most from a bioelectronic approach (Figure 3).

## 6 Challenges and future perspectives on the use of CSN neuromodulation to treat type 2 diabetes

Whereas the CSN represents an electrical neuromodulation target for treating T2D, several challenges need to be addressed to provide a personalized, adaptable and real-time treatment with minimal side effects. The interface between a neural device and the peripheral nerves is guided by several requirements in terms of design and materials, both contributing to the foreign body response (FBR) of the device. The FBR leads to encapsulation of the device by fibrotic tissue, that behaves as an electrical insulator reducing the electrical performances of the electrodes (Anderson et al., 2008). Surface biocompatibility is a crucial factor for designing a neural electrode, involving chemical, physical, morphological, and biological interactions of a device with the surrounding tissues. Moreover, the mechanical properties and the shape of the device also influence the degree of severity of the FBR. Typically, the use of soft inert materials is preferred for neural electrode, to reduce both the mechanical and chemical mismatch between the device and the nerves (Paggi et al., 2021). Moreover, the device should be miniaturized to avoid damaging of the surrounding tissues and increase selectivity. Biocompatibility requirements are closely linked to the performance of the electrodes. To achieve better results in terms of recording and stimulation capabilities, the electrical impedance should be low, but there is a trade-off to consider between size and impedance, because the latter increases when the size decreases (Boehler et al., 2020). The overall design of neural electrodes, therefore, should be aimed at reducing the FBR increasing at the same time the selectivity in terms of recording and stimulation. This would allow to selectively modulate only the interested neural population, enhancing the neuromodulation performances and reducing in turn the adverse effects.

The choice of electrode materials is also influential in the implementation of the KHFAC. Delivering current at high frequency (1–60 kHz) can result in electrode polarization and contamination of direct current (Ghazavi and Cogan, 2018; Patel et al., 2018). Typical electrode materials are platinum (Pt), titanium nitride (TiN), and sputtered iridium oxide (SIROF), that rely on different mechanisms for charge injection (Faradaic, non-reversible, and non-Faradaic, reversible). These materials have been tested to evaluate the block threshold (BT) and the onset response (BR); the two main parameters involved in KHFAC. Both of them should be minimized: the block threshold can be defined as the amplitude value completely blocking the nerve conduction, while the onset response as the initial nerve activity starting after the block is imposed (Green et al., 2022). Patel et al. (2018) evaluated different electrode materials among the most commonly used, without identifying any significant difference in BT and OR. This could be due to the frequency range used for the stimulation: at higher frequency (20–60 kHz), the electrochemical characteristics become independent from the electrode material, while at lower frequency (1–5 kHz) Pt electrodes suffer from a significant polarization effect (Ghazavi and Cogan, 2018). An interesting approach has been pursued by Green et al. (2022) using carbon black coatings, that significantly decreased BT for a wide range of frequency. This is due to its high capacitance properties, that enhance the possibility of safely deliver charge.

Another important issue to overcome is the chronicity of high frequency stimulation aiming at the minimization of CSN neuromodulation off-target effects. The continuous CSN KHFAC modulation simulates a CSN resection procedure and therefore, as discussed earlier, might produce several side effects related with the abolishment of CB physiological actions, as the loss of responses to hypoxia, fluctuations in blood pressure and impaired responses to exercise (Conde, 2017). Minimization of CSN neuromodulation off-target effects can be achieved using closed-loop feedback control systems. Closed-loop devices have been successfully developed to improve therapeutic efficacy, streamlining clinic workflow, and increase the quality of life for patients for different therapeutic applications including regulating heart rate (Biotroniks pacemakers) and controlling glucose levels (artificial pancreas). However, closed-loop systems for delivering therapeutic patterns of electrical stimulation to peripheral nerves in T2D, have never been established. Advances in bioelectronic medicine towards these closed-loop systems are strengthened by the development of new implantable electrodes and algorithms, enabling a safe, effective, and minimally invasive interface with the nervous system (Rijnbeek et al., 2018; Zhou et al., 2018). Therefore, CSN neural decoding and characterization of high frequency stimulation paradigms that reverse metabolic dysfunction would provide key information to combine recording and stimulation modalities for closed-loop applications to correct altered CSN neural patterns on demand. This closed-loop approach will offer tailored CSN bioelectrical neuromodulation that will bring significant advantages compared to current, depersonalized, lifestyle management, pharmacological or surgical options for this disease.

Future research directions are also related with the hardware solutions allowing the power supply to the high frequency stimulation of the CSN (Burton et al., 2021). These energy requirements for KHFAC dictate three things: the amount of power needed, the surgery and size of incision to place the power supply, and the patient recharge adherence. Power supply can be enormously reduced using closed-loop systems, allowing the delivery of electrical current only when needed, however other options are being developed to decrease energy requirements including the internal body-generated power (Bullen et al., 2006; Settaluri et al., 2011; Hwang et al., 2015; Yao et al., 2018) the use of external power supply obtained through via radiofrequency electromagnetic fields (Gutruf et al., 2019), light illumination (Ding et al., 2018) or ultrasonic waves (Charthad et al., 2015). These external sources produce significant amounts of energy (~500 mW) that can be consistently transmitted, with versatile design and deployment options (Yu et al., 2019). Secondly and in relation to the location of the power supply within the body, implantable pulse generators positioned subcutaneously in different body locations, such as clavicle or axilla, have been shown to have good safety and tolerance for VNS in epilepsy patients (Sakas et al., 2007), for deep brain stimulation in Parkinson disease (Frey et al., 2022) and psychiatric disorders (Jakobs et al., 2023), and therefore can be an option to be used for KHFAC CSN stimulation. Thirdly, and thinking on the recharging requirements, even if needed multiple times a day, would perhaps still be less burdensome than some T2D therapeutic options such as the multiple insulin injections (Won et al., 2023).

A multitude of opportunities is also related to the use of CSN activity indirect assessment as a screening tool, not only for diabetes, but also for obesity (Sacramento et al., 2020), resistant hypertension (Young et al., 2009), heart failure and sleep apnea (Dunn et al., 2018). Screening tests are performed in asymptomatic patients aiming to detect diseases at an early stage before any symptoms become apparent. CB activity screening should be offered as an opportunistic screening, performed during yearly routine visits to the General Practitioners as a population-wide screening operation or directed to the population with significant risk factors for diabetes like family history, obesity or overweight, age above 45 years, sedentarism, gestational diabetes and/or macrosomic siblings, sleep quantity/quality, ethnicity among others (Ding et al., 2018). The screening of CB activity would allow early detection of subclinical sympathetic overactivation, and monitoring of the effectiveness of lifestyle intervention approaches. Presumably, the population which will benefit the most from CB screening are the siblings of metabolic patients that will have access to a quantifiable non-invasive indirect assessment of sympathetic status which will widen the spectrum of preventive medicine approaches. Also, for patients with T2D diagnosis, screening of CB activity will allow assessing if each individual patient is a suitable candidate for treatment with CB neuromodulation. One of the constraints to the implementation of this measure is the cost of implementation on the ground and the burden that another screening program of this size may bring to the already overburdened local and national health services. The exponential growth of the wearables industry may represent an opportunity to be explored for this purpose, with the CBmeter prototype representing a first pilot approach. Provided they are validated, sensors that monitor the parameters of interest will allow health monitoring outside of the clinic, generating information that properly analyzed, may represent a new way to screen for CB overactivity and to predict health events (Dunn et al., 2018). All these considerations require substantial work to be tested and validated. The future research directions include performing a longitudinal clinical study to assess the prognostic value of CB activity assessment for T2D diagnosis. Feasibility studies for the clinical efficacy if CSN neuromodulation in humans will also be required, in line with what has been done in the last couple of years with vagal stimulation in rheumatoid arthritis (Kanashiro et al., 2018). After clinical validation, these new neuromodulatory algorithms have the potential to be innovative approaches and an alternative to classical drug-based therapies.

## Author contributions

SC: Conceptualization, Writing – original draft, Writing – review & editing. JS: Writing – original draft, Writing – review & editing. CZ: Writing – original draft, Writing – review & editing. AM: Writing – original draft, Writing – review & editing. SM: Writing – original draft, Writing – review & editing. MG: Writing – original draft, Writing – review & editing.

## Footnotes

^1^ https://clinicaltrials.gov/study/NCT05003310
